# 

**Published:** 1912-12-15

**Authors:** 


### ANNOUNCEMENTS.

Chicago Dental Society.—The officers of the Chicago Dental Society are planning a large celebration for Friday and Saturday, January 31, and February 1, 1913. The program includes two days of clinics by selected men from all parts of the country, one evening of papers by men of international reputation, concluding the two days' meeting with a testimonial banquet to our esteemed confrere, Dr. Truman W. Brophy of Chicago. The dentists of Chicago will make every effort to see that the entire program will eclipse all former meetings. Any dentist who has a new or interesting clinic to give at this meeting is cordially invited to correspond with the chairman of the Clinic Committee, Dr. Fred. W. Gethro, 917 Marshall Field Bldg., Chicago, Ills.

---

Institute of Dental Pedagogics. The next annual meeting of the Institute of Dental Pedagogics will be held in Pittsburgh, Pa., January 28, 29 and 30, 1913. An unusually interesting program has been arranged and no progressive dental teacher can afford to miss this meeting.

Fred. W. Gethro, *Secretary*.

